# Structure design method of new balanced vibration reduction gear for the three cylinder engine

**DOI:** 10.1371/journal.pone.0266560

**Published:** 2022-04-13

**Authors:** Pingjun Wang, Gangyan Li, Sirui Liu, Xiaoxu Wei

**Affiliations:** 1 College of Mechanical and Electrical Engineering, Wuhan University of Technology, Wuhan, China; 2 College of Automotive and Electromechanical Engineering, Xinyang Vocational and Technical College, Xinyang, China; National Textile University, PAKISTAN

## Abstract

Aiming at the engineering requirements of reducing the volume and improving the vibration characteristics of gears in the three-cylinder engine balanced system, a design and optimization method of gear structure is proposed based on the Design of Experiments (DOE) and proxy models. The paper analyzes the structure improvement process based on the gear design model and technical index requirements. By designing the plane characteristics of the weight-increasing module, the weight-reducing module and the elastic module, the calculation model of balance performance indices such as mass, moment of inertia and unbalance of new balanced vibration reduction gear are constructed. Then, a more efficient design method is proposed based on dynamic simulation and multidisciplinary optimization design platform (Isight). The results show that the new design method of gear structure can effectively reduce the structure improvement cycle. At the same time, the improved structure can reduce the thickness of the weight-increasing module by 6.3 mm and the vibration attenuation by more than 90%.

## Introduction

With the demand for energy savings and emission reduction of automobiles, how to solve the vibration problem based on meeting the requirements of lightweight and high performance is an important problem faced by the three-cylinder engine. The congenital structure of the three-cylinder engine causes an imbalance in the first-order reciprocating moment of inertia. To offset the imbalance, a counterweight is often used. The balance shaft counterweight is a common mechanism to reduce the engine’s moment of inertia to meet engine vibration damping and noise reduction requirements [1–3]. In the existing three-cylinder engines equipped with balance shafts, the gear driving method is often used. Factors such as the structure shape, material, mass, and inertia of the gears directly affect the vibration damping performance of the balance shaft, and the utilization of the assembly space of the balanced system is considered. The design of the balance mechanism is required to reduce the number and volume of parts as much as possible [4]. In engineering applications, the lack of theoretical support for the structural design of the balanced vibration reduction gearhas led to problems such as long product improvement design cycles and imperfect design schemes. Therefore, research on structural design method of the gear in the three-cylinder engine balanced system has practical engineering significance.

Many scholars have performed research on the gear structure of balanced system. A typical example is Trieschmann J et al. [5], who invented a steel gear for the balance shaft. It was proposed that the performance of the gear material will affect the function and durability of the balance shaft. Moetakef MA [6] invented a gear for balance shafts. He also proposed that the noise generated by meshing gears is related to the material, mass, inertia and other factors. The design of elastic blocks overcame the noise, vibration and other problems of traditional rigid gears. Alessio Courtial et al. [7] invented a counterweight gear for a balance shaft, using the principle that the balanced block and the gear rotate together to provide the engine with a counterrotating force to balance the reciprocating inertial force of the engine. Ishikawa M et al. [3] compared and analyzed the balance shaft performance characteristics of a 2AZ-FE engine with elastic gears and a Toyota 5S-FE engine with rigid gears. The application of elastic gears could increase the cost and friction of the balance shaft system. The weight was reduced by 50%, and the gear transmission noise and vibration could be reduced at the same time. Wen [8], Renping S [9], and Singh OP [10] analyzed the dynamic response of elastic teeth and gear bodies and obtained the structural design of elastic gears to reduce the transmission load and prolong the service life. Gao [11], Wehrle E, et al. [12] constructed the parametric design formulas of the gear counterweight module and obtained the theoretical calculation formulas of vibration force and moment. By optimizing the gear counterweight mass, the vibration behavior of the gear drive was improved.

For the structural design method of vibration balanced gear, Monkova K et al. [13] discussed the influence of gear weight reduction on its modal characteristics. They introduced the dependence of natural frequency on the change of gear shape by using simulation tools. By designing the damping ring structure, Wang [14] reduced the axial vibration of the gear and obtained the influence of the friction coefficient and mass of the damping ring on the damping characteristics. Kumar S M et al. [15] improved the gear transmission efficiency by removing the gear material and found that the weight reduction method with a circular shape was more effective. Geng et al. [16] studied the influence of the damping coefficient of the damping ring on gear transmission vibration by applying the dynamic analysis method. To further optimize the influence of the structural parameters of the gear on its vibration-damping performance, some scholars analyzed the optimization method of the vibration balanced gear structure. Han et al. [17] proposed a new optimization model that can simultaneously carry out the optimal design of size constraints and layout constraints for arbitrary gear transmission. Kim J et al. [18] optimized gear webs for rotorcraft engine balanced gear trains by the NSGA optimization method. Qi et al. [19] and Xu et al. [20] studied the application of the response surface method in the structural optimization of gear transmission and damping ring design. Freddy et al. [21–23] analyzed the specific application of the response surface method in the structural optimization method.

The above analysis showed that the key points of the structural design of the gears of the three-cylinder engine balanced system were the design of the damping module and the optimization of the counterweight module. The balance performance index was taken as the constraint. By constructing the parametric design formulas of the balanced system gears, multiple objectives were applied. The optimization method to analyze the key structure parameters of the gears was implementable. Therefore, the design strategy in this paper was aimed at the structural design and optimization method of the most important balanced vibration reduction gearin the balanced system. The second part of the paper expounded on the balance performance requirements and structure improvement requirements of the three-cylinder engine balanced system. The third part proposed the improvement design method and specific implementation steps of the structure of balanced vibration balanced gear. Finally, we completed the verification analysis of the optimized structure of the engagement impact performance and vibration by dynamic simulation.

## Materials and methods

### Problem statement

**Fig 1:**
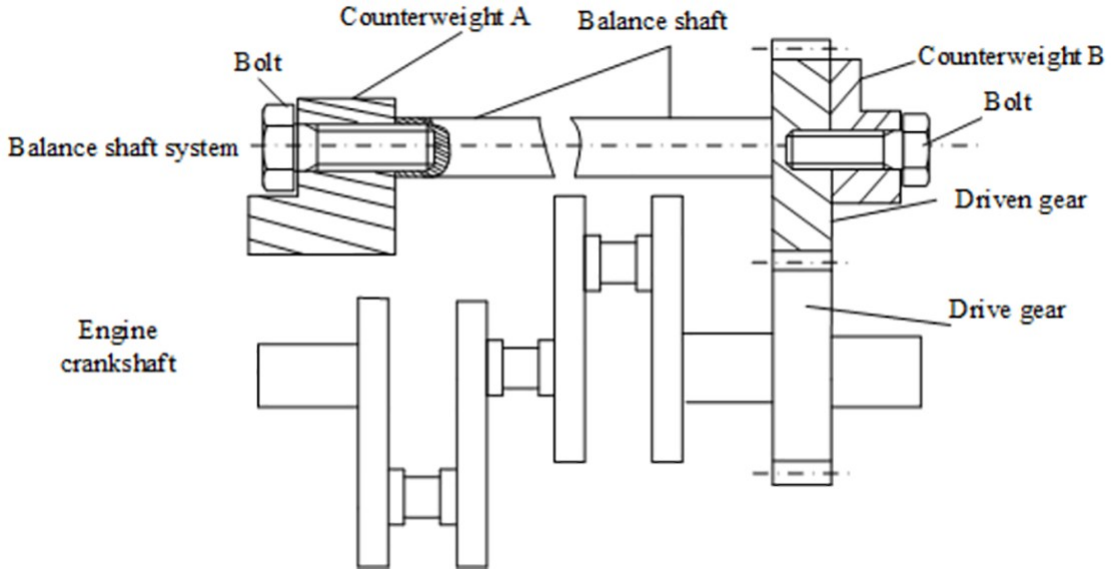
Schematic diagram of the balanced system structure in the three-cylinder engine.

The three-cylinder engine balanced system is composed of balance shaft, counterweights, gears, bearings and other components. Through gear meshing transmission, the power of the engine crankshaft is transmitted to the balanced system. With the help of the counterweight module in the balanced system, part of the three-cylinder is offset. Hence the unbalanced torque is produced by the rotation of the engine’s crankshaft. The structure of a certain type of three-cylinder engine balanced system is shown in Fig 1.

**Fig 2:**
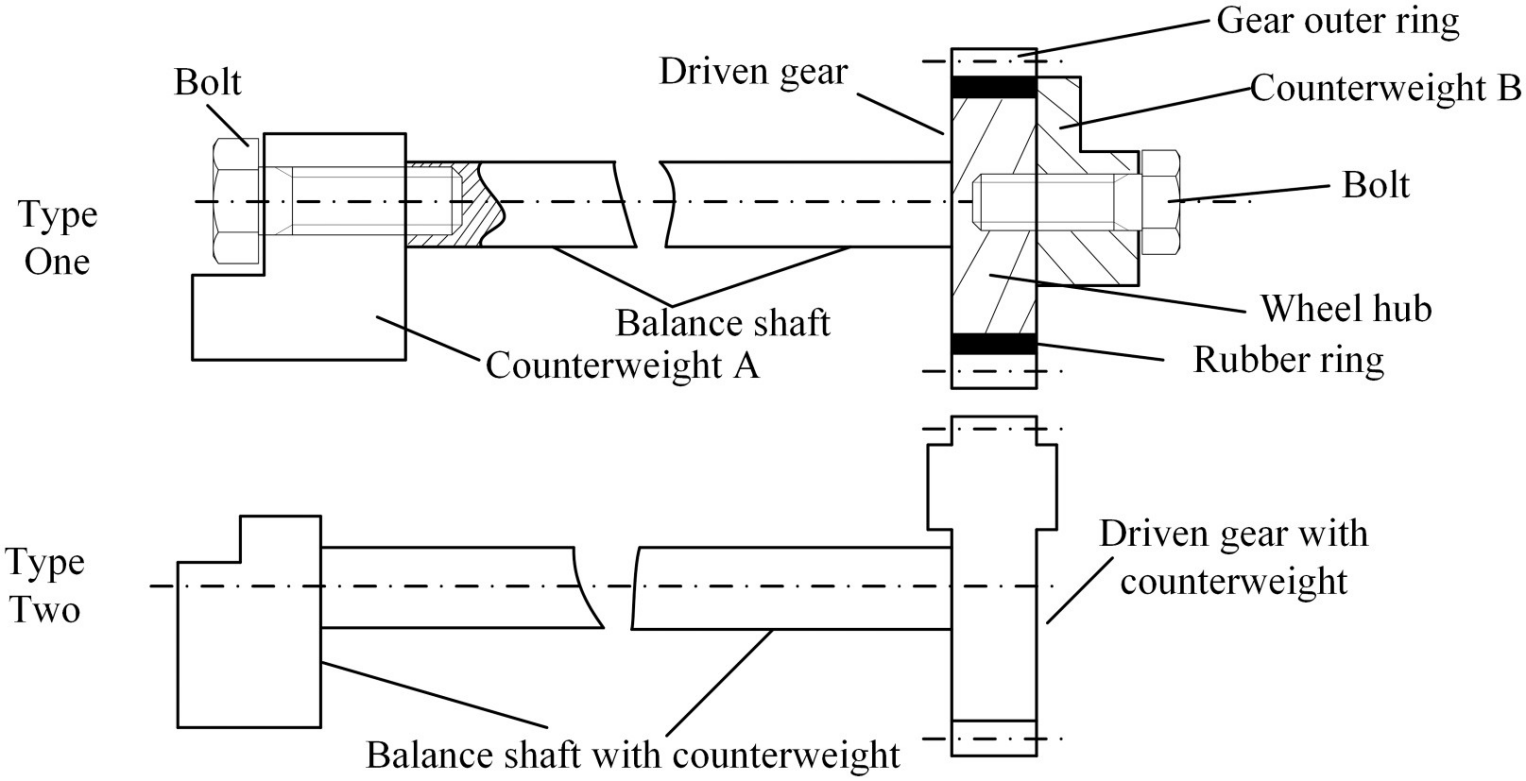
The structure of three-cylinder engine balanced system.

The shape and position of the counterweight of the three-cylinder engine balanced system have a certain effect on the balance of the inertial force and moment of the engine. Fig 2 shows the structure of the gears in the three-cylinder engine balanced system.

It can be seen that making the counterweight gear into an integrated structure can improve the assembly efficiency of parts and components, make the overall structure of the three-cylinder engine balanced system short, and facilitate the layout of the whole machine. However, because the counterweight part is located in the gear plate and there is no design of damping material in the counterweight gear structure, it is easy to produce a large instantaneous meshing impact during the movement. The elastic gear described in type one will reduce meshing impact and vibration.

**Fig 3:**
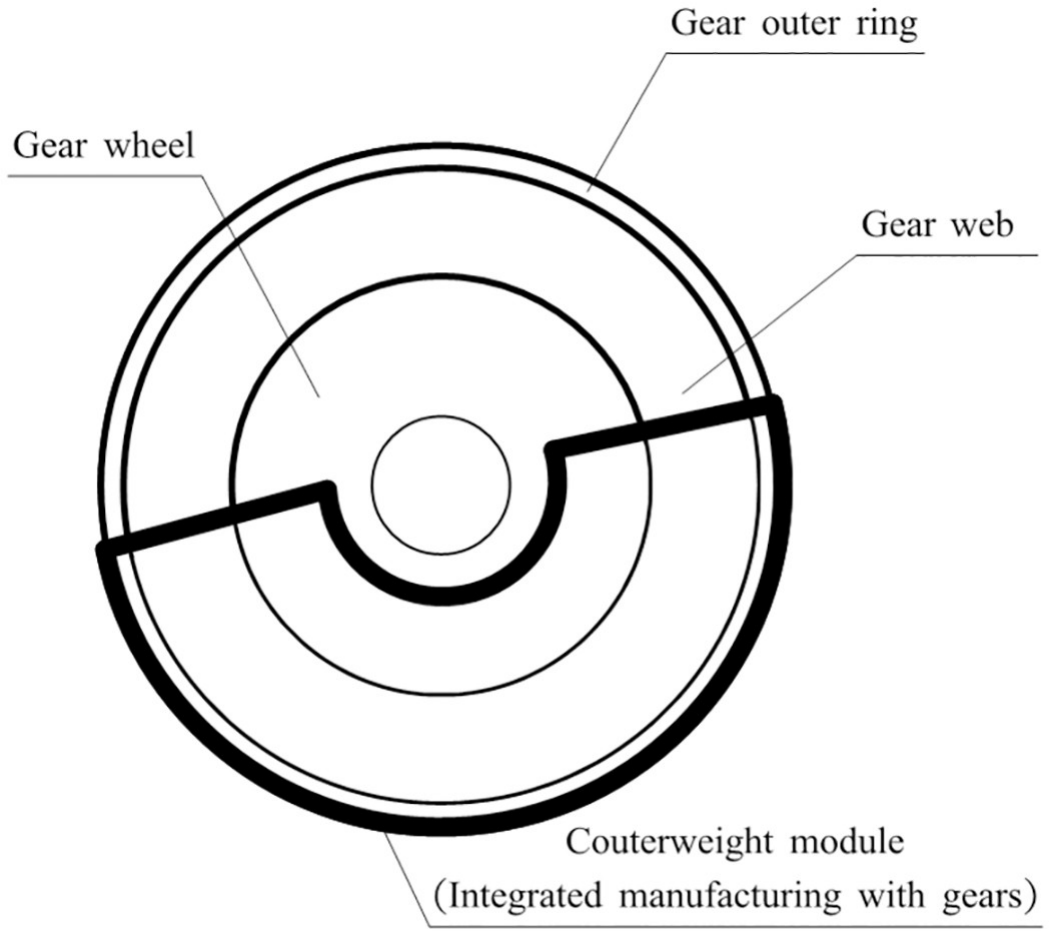
The structure of driven gear in the three-cylinder engine balancing system.

Fig 3 shows the structure diagram of the driven gear of a certain type of three-cylinder engine balanced system that needs to be improved.

The engineering requirements for specific gear improvements are as follows: (i) Considering the improvement of its vibration-damping performance, the design requirements of increasing the elastic module are proposed. (ii) Considering the requirements of lightweight and compact size, the requirements of reducing the thickness of the counterweight module are proposed. In response to the above requirements, combined with the characteristics of elastic gears and counterweight gears, this paper proposes a balanced vibration reduction gearstructure that integrates the elastic module and the balanced weight module. At the same time, considering the limited assembly space of the counterweight gear with only the weight-increasing module, the design of the weight-reducing module is proposed to ensure the requirements of the lightweight and small size of the gear.

### Structural optimization

**Fig 4:**
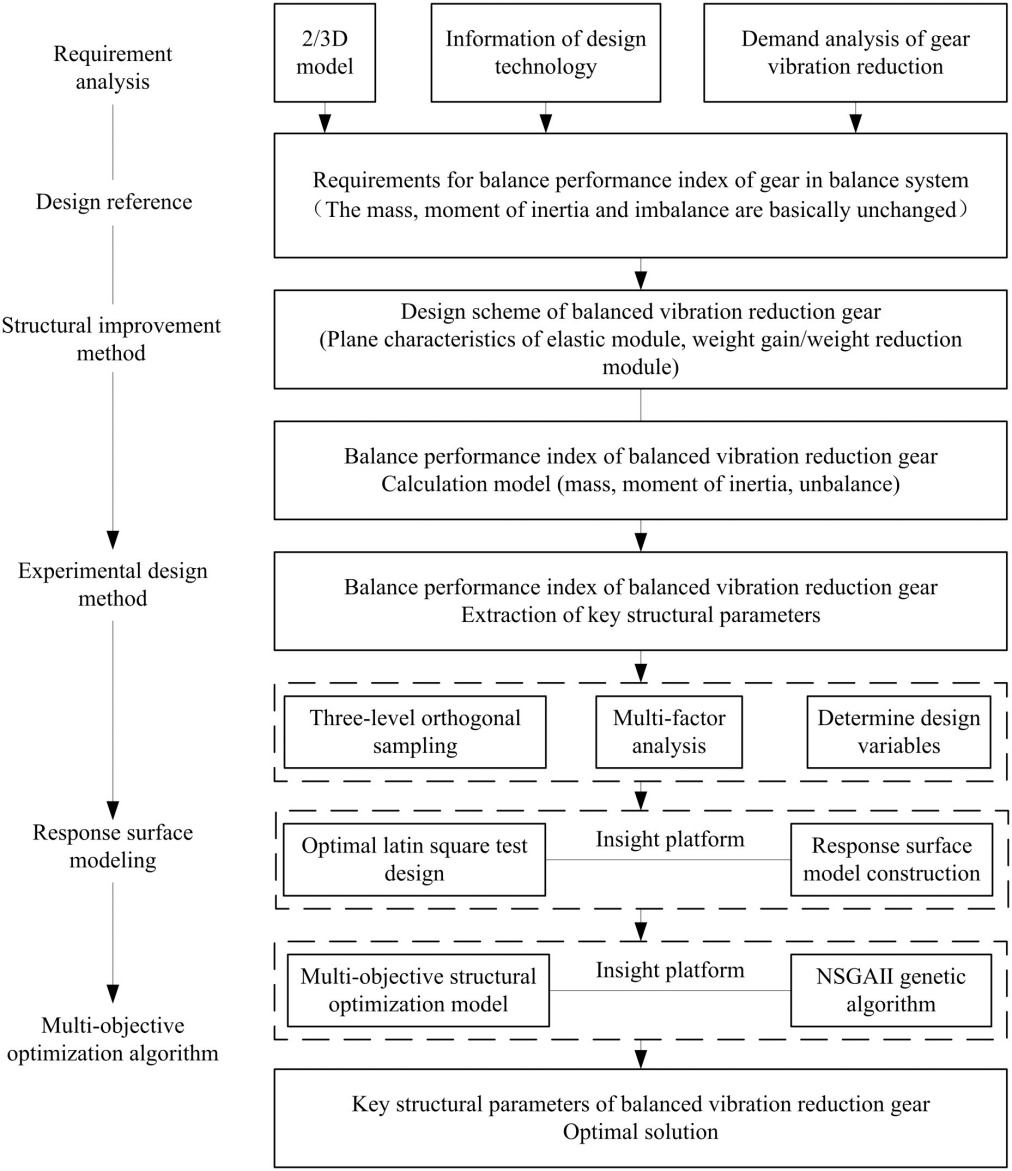
Design process for improving the structure of the balanced vibration balanced gear.

To ensure the effect of the balanced system in three-cylinder engine, it is necessary to ensure that the balance characteristics of the gear. The balanced characteristics of the gear include mass, moment of inertia and imbalance. The geometric shape and material density changes of the balanced vibration reduction gearwill affect its mass, moment of inertia, and imbalance. Therefore, the structural design of the balanced vibration reduction gearis carried out under the condition that the mass, moment of inertia and unbalance of the improved gear remain unchanged in this paper. And this paper proposes a structural design method based on DOE and the agency model. Fig 4 shows the improvement design process of the structure of the gear in three-cylinder engine balanced system.

The specific implementation steps are as follows.

1. Based on the 2D, 3D models and technical data of the three-cylinder engine balanced system, the design requirements for the balanced performance index are extracted. The specific index range is shown in Table 1.
2. Based on the principle that the balance performance indicators (mass, moment of inertia, unbalance) are unchanged, the plane characteristics of the weight increasing module, elastic module, and weight reduction module are proposed.
3. Based on the structural characteristics of the proposed balanced vibration reduction gearand applying basic mathematical theory, a calculation model of its balanced performance index is obtained.
4. Apply the DOE experimental design method to extract key structure parameters.
5. Apply proxy model to improve the efficiency of structure optimization.
6. Taking the mass, moment of inertia, imbalance and design range of various parameters as basic constraints, a multiobjective optimization algorithm is applied to obtain the optimal solution of key parameters.
7. Based on the simulation technology, the rationality of the optimized structure is verified.

**Table 1:** Balance performance index of a certain type of three-cylinder engine balanced system.

Part name	Material	Mass /g	Moment of inertia / g∙cm^2^	Static imbalance / g∙cm	Working conditions and performance requirements
Balance shaft with counterweight	50CrMo4	1304.7	3223.8	487.6	800~6600rpm
Gear with counterweight	16MnCr5HL	664±10	12179 ±5%	491.5±5	The radial clearance is 4.9 mm, and the axial clearance is 2.1 mm.

### Mathematical model

To facilitate the calculation and analysis of balance performance indicators such as the mass, moment of inertia, and imbalance of the balanced vibration balanced gear, the key parameter symbols and meanings can be found in Table 2.

**Table 2:** Main parameters of the balanced vibration balanced gear.

Parameter	Significance	Unit
*R*	Inner radius of weight-increasing module	*cm*
*L*	The distance from the intersection of the inner circle and cross section of the boundary of counterweight to the X axis	*cm*
*θ*	The angle between the cross-section of the boundary of the counterweight and the X axis	°
*H*	Weight-increasing module thickness	*cm*
*d*_*k*_	Weight reducing hole diameter	*cm*
*β*_2_	The angle between the weight-reducing holes	*cm*
*h*_*c*_	Depth of weight reduction tank	*cm*
*b*_*c*_	Width of weight reduction tank	*cm*
*2γ*	The fan-shaped part of the weight reduction groove corresponds to the expansion angle	°
*R*_*x*_	Rubber ring outer diameter	*cm*
*h*_*x*_	Rubber ring thickness	*cm*
*m*_*i*_*(i = p*,*k*,*c*,*x)*	The mass of each module of vibration balanced gear	*g*
*J*_*i*_*(i = p*,*k*,*c*,*x)*	Rotational inertia of each module of vibration balanced gear	*g·cm* ^*2*^
*U*_*i*_*(i = p*,*k*,*c*,*x)*	Unbalance quantity of each module of vibration balanced gear	*g·cm*
*ρ*_*i*_*(i = p*,*c*,*x)*	The density of each module of vibration balanced gear	*g/cm* ^*3*^

According to the plane characteristics of the balanced vibration balanced gear, the calculation method of the volume of irregular objects is used, and the theoretical calculation basis of mass, moment of inertia, and unbalance are applied to obtain the weight increasing module, weight reduction hole module, and weight reduction slot module of the balanced vibration balanced gear.

**Fig 5:**
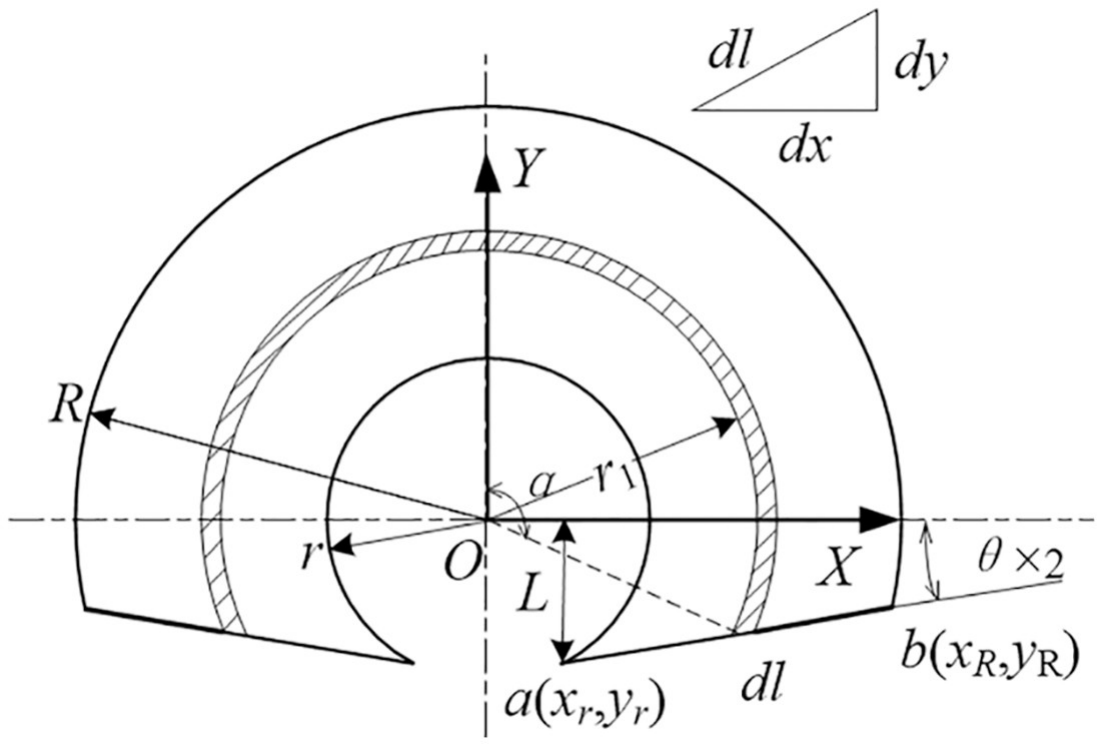
Plane characteristics of weight-increasing module.

Fig 5 indicates the schematic diagram of weight-increasing module calculation, the transversal on the right side of the Y-axis is expressed as Formula 1.


$$y=\text{tan}\;\theta \cdot x-\sqrt{r^{2}-L^{2}}\;\text{tan}\;\theta \text{+}L$$ (1)

Then the x-coordinate expression of the two intersecting points *a* and *b* is shown in Formula 2

$$\left\{\begin{array}{c} x_{r}=\sqrt{r^{2}-L^{2}}\\ x_{R}=\frac{-bk+\sqrt{b^{2}k^{2}-(1+k^{2})(b^{2}-R^{2})}}{1+k^{2}} \end{array}\right.$$ (2)

Where *k* and *b* are the slope and vertical intercept of the transversal respectively, which is shown in Formula 3.


$$\left\{\begin{array}{c} k=\text{tan}\;\theta \\ b=-\sqrt{r^{2}-L^{2}}\;\text{tan}\;\theta \text{+}L \end{array}\right.$$ (3)

The final result is obtained by integration method as shown in Formula 4 [24, 25], where *e*_c_ is the eccentric distance and *I* is the arc length, which is expressed as Formula 5.


$$\left\{\begin{array}{l} m_{p}=\int dm=\int_{x_{r}}^{x_{R}}\rho_{p}HI\cdot \sqrt{1+k^{2}}dx\\ J_{p}=\int r_{1}{}^{2}dm=\int_{x_{r}}^{x_{R}}r_{1}{}^{2}\rho_{p}HI\cdot \sqrt{1+k^{2}}dx\\ U_{p}=\int e_{c}dm=\int_{x_{r}}^{x_{R}}e_{c}\cdot \rho_{p}HI\cdot \sqrt{1+k^{2}}dx \end{array}\right.$$ (4)

$$I=(\text{arctan}\frac{x}{kx+b})\sqrt{x^{2}+{\left(kx+b\right)}^{2}}$$ (5)

**Fig 6:**
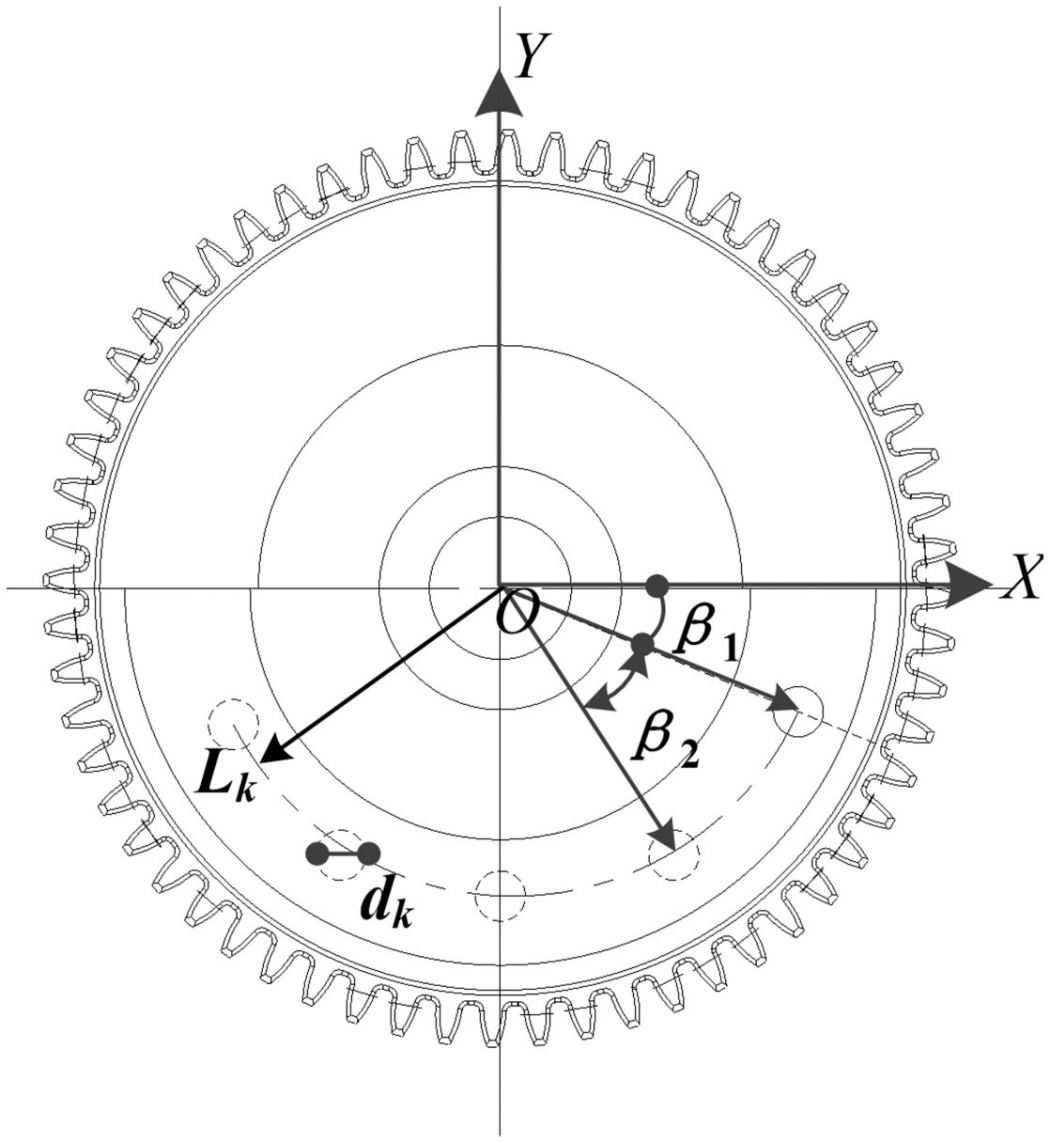
Plane characteristics of weight-reducing hole module.

Fig 6 indicates the schematic diagram of weight-reducing hole module calculation. Assuming that the hole is solid, the hole depth is *h*_k_ and the number of designed holes is *n*. The calculation result of the balance characteristic index of the weight-reducing hole module is shown in Formula 6 [24, 25].


$$\left\{\begin{array}{l} m_{k}=nL_{k}\pi \frac{d_{k}{}^{2}}{4}h_{k}\\ J_{k}=nm_{k}{\left(\frac{d_{k}}{2}\right)}^{2}+nm_{k}{\left(L_{k}\right)}^{2}\\ U_{k}=m_{k}y_{k} \end{array}\right.$$ (6)

**Fig 7:**
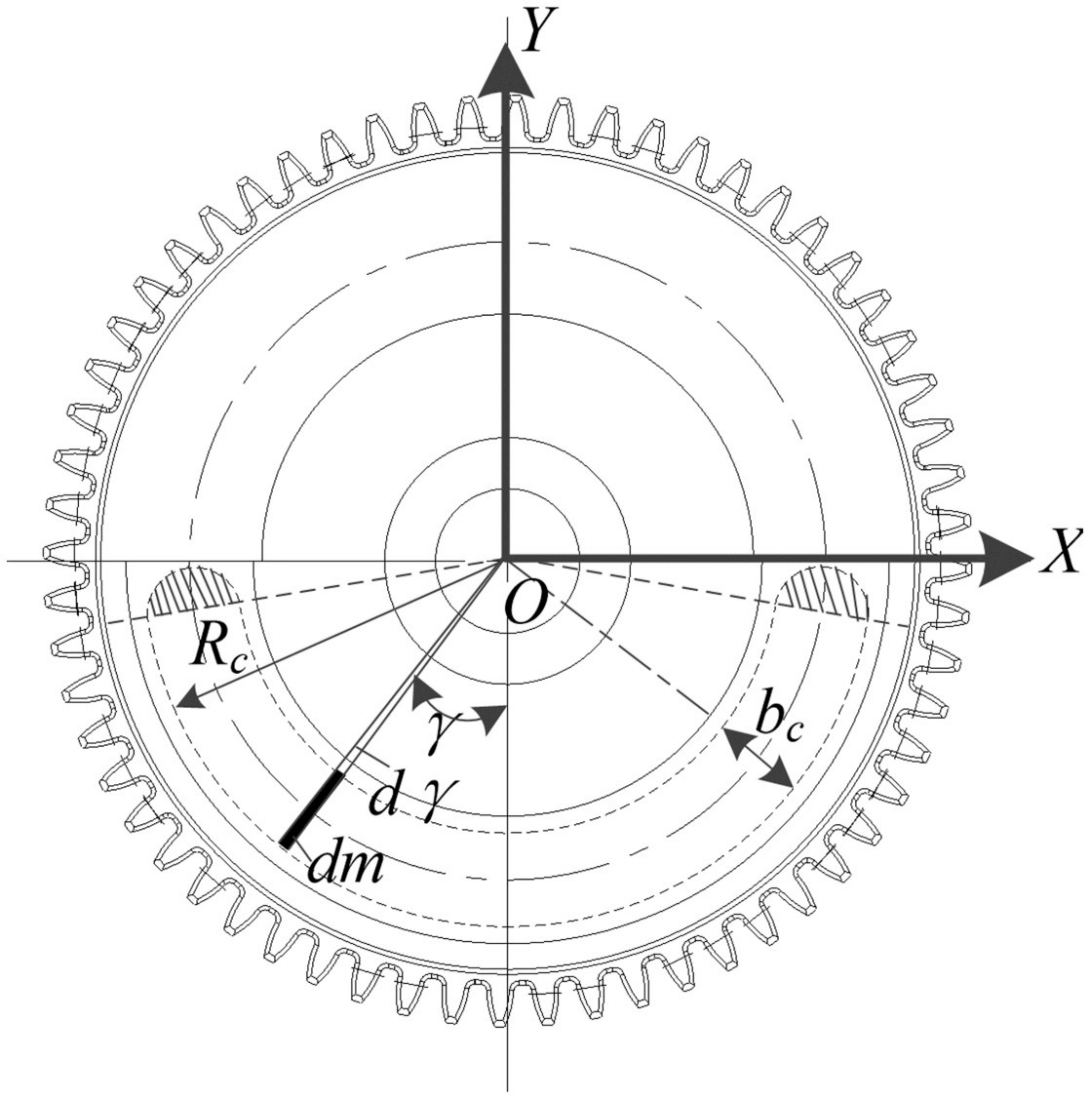
Plane characteristics of weight-reducing groove module.

The schematic diagram of weight-reducing groove calculation is shown in Fig 7. The calculation result of its balance characteristic index is shown in Formula 7 [24, 25], where *y*_c_ is the y-coordinate of the center of mass of the half disk.


mc=2bcRc−bc2hcρcγ+14πbc2hcJc=2bcRc−bc2hcρcγRc2+172π2πbc2hcr2c(9π2−32)+14πbc4hcUc=2bcRc-bc2hcρcRcsinγ+12πbc2hcyc (7)

**Fig 8:**
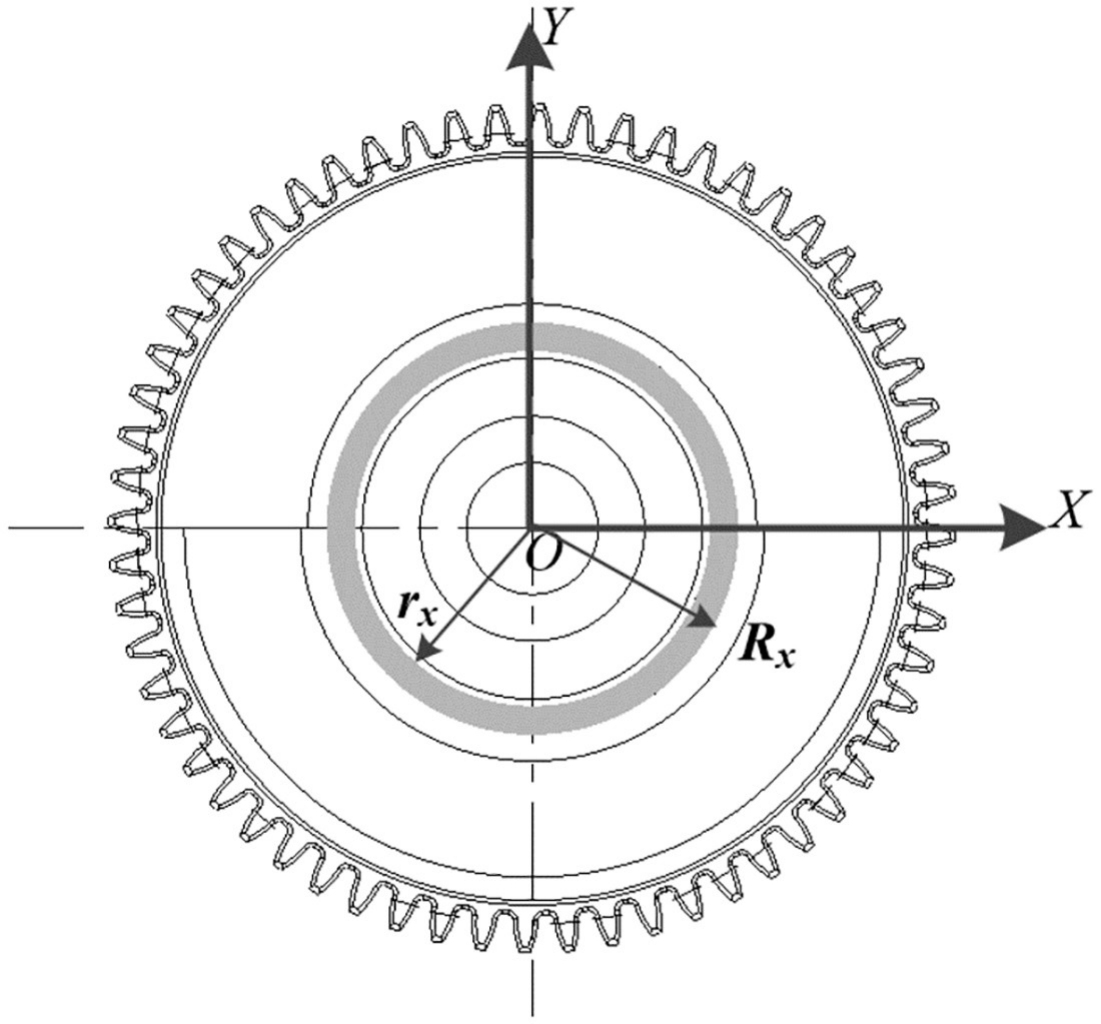
Plane characteristics of rubber ring module.

Combined with Fig 8, the calculation result of the balance characteristic index of the elastic module is shown in Formula 8 [24, 25].


$$\left\{\begin{array}{l} m_{x}=\rho_{x}\pi (R_{x}{}^{2}-r_{x}{}^{2})h_{x}\\ J_{x}=\frac{1}{2}m_{x}(R_{x}{}^{2}+r_{x}{}^{2})=\frac{1}{2}\rho_{x}\pi (R_{x}{}^{2}-r_{x}{}^{2})h_{x}(R_{x}{}^{2}+r_{x}{}^{2})\\ U_{x}=0 \end{array}\right.$$ (8)

Because of the gear teeth, webs, and hub parts of the balanced vibration reduction gear are the standard structure, the influence of changes in the structure parameters on the calculation of the balanced characteristic index is not considered. Therefore, the balanced characteristic index of the three-part structure is a fixed value. Combining the calculation model of the balance performance index of the above modules, the balanced characteristic index of the gear is shown in Formula 9.


$$\left[\begin{array}{c} m\\ J\\ U \end{array}\right]=\left[\begin{array}{c} m_{T}\\ J_{T}\\ U_{T} \end{array}\right]+\left[\begin{array}{c} m_{W}\\ J_{W}\\ U_{W} \end{array}\right]\text{+}\left[\begin{array}{c} m_{H}\\ J_{H}\\ U_{H} \end{array}\right]\text{+}\left[\begin{array}{cccc} 1 & -1 & -1 & 1\\ 0 & 0 & 0 & 0\\ 0 & 0 & 0 & 0 \end{array}\right]\left[\begin{array}{c} m_{p}\\ m_{k}\\ m_{c}\\ m_{x} \end{array}\right]+\left[\begin{array}{cccc} 0 & 0 & 0 & 0\\ 1 & -1 & -1 & 1\\ 0 & 0 & 0 & 0 \end{array}\right]\left[\begin{array}{c} J_{p}\\ J_{k}\\ J_{c}\\ J_{x} \end{array}\right]+\left[\begin{array}{cccc} 0 & 0 & 0 & 0\\ 0 & 0 & 0 & 0\\ 1 & 1 & -1 & 1 \end{array}\right]\left[\begin{array}{c} U_{p}\\ U_{k}\\ U_{c}\\ U_{x} \end{array}\right]$$ (9)

### Parameter selection

In the calculation model of the balanced performance index of the balanced vibration balanced gear, there are more than a dozen structure parameters that affect its quality, moment of inertia, and imbalance. The DOE experimental design method is one of the statistical methods to analyze the relationship between the key structure parameters and performance indicators of the product and to determine the optimal parameter combination. It has been widely used in structural design optimization [26, 27]. This method was adopted to obtain the key structure parameters that affect the balance performance index of the balanced vibration reduction gear and to determine the optimal value. The orthogonal experimental design method was adopted to analyze the key structural parameters.

Considering the constraints and correlations between the structure parameters, 11 structure parameters were selected as the analysis variables of the orthogonal experimental design. According to the economy and efficiency of machining and the structural parameters of the balanced vibration balanced gear, three levels were selected for each factor. The difference between the same factors and different levels is the same. The valuse of selected levels are shown in Table 3.

**Table 3:** Factor level table.

Level	Factor										
	*R* (cm)	*L* (cm)	*θ*(°)	*H*(cm)	*d*_*k*_ (cm)	*β*_2_(cm)	*h*_*c*_ (cm)	*b*_*c*_ (cm)	*γ*(°)	*R*_*x*_ (cm)	*h*_*x*_ (cm)
1	3.0	3.0	0°	0.425	0.375	30°	0.2425	0.525	0°	2.3	0.95
2	3.75	1.5	45°	0	0.45	37.5°	0	0.7625	45°	2.475	0.7175
3	4.5	0	90°	0.85	0.525	45°	0.485	1.0	90°	2.65	0.485

According to the results of the orthogonal experiment, the balanced characteristic were calculated, and the calculation results were processed by the averaging method to obtain the comprehensive score value of each test. Based on the principle of range analysis and the calculation results of the performance indicators, the order of the degree of influences of the key structure parameters on the comprehensive balanced characteristic indicators is

$$R>\theta >H>b_{c}>\beta_{2}>d_{k}>L>h_{x}>\gamma >R_{x}>h_{c}$$ (10)

It can be obtained from Formula 10, rubber ring thickness *h*_*x*_, rubber ring outer diameter *R*_*x*_, depth of weight reduction tank *h*_*c*_, the fan-shaped part of the weight reduction groove corresponds to the expansion angle *γ* have relatively little influence on the comprehensive equilibrium characteristics. Therefore, inner radius of weight-increasing module *R*, the distance from the intersection of the inner circle and the cross section of the boundary of the counterweight to the X axis *L*, the angle between the cross-section of the boundary of the counterweight and the X axis *θ*, weight-increasing module thickness *H*, weight reducing hole diameter *d*_*k*_, the angle between the weight-reducing holes *β*_*2*_, width of weight reduction tank *b*_*c*_ are taken as optimization variables, the design variables that form structure parameter optimization are shown in Formula 11, where *x*_i_(i = 1,2,3,4,5,6,7) represent *R*, *θ*, *L*, *H*, *d*_k_, *β*_2_ and *b*_c_ respectively.


$$x={\left[x_{1},x_{2},x_{3},x_{4},x_{5},x_{6},x_{7}\right]}^{T}$$ (11)

### Mapping model

The radial basis function can effectively construct the invisible relationship between input and output [28]. The response surface method [29] can reduce the number of experiments and improve the overall optimization efficiency. To fully and accurately reflect the actual relationship between the key structure parameters and the mass, moment of inertia, and unbalance, the radial basis function response surface method was used to establish the mapping model of key structure parameters and balanced characteristic indicators.

The experimental design adopted the optimal latin hypercube design method. According to the input range of the design variables in Table 1, 36 sets of response design schemes were carried out on the 7 structural design parameters of the balanced vibration balanced gear.

A program integration component (Simcode) under the test design component in the optimization design simulation integrated platform (Isight) was established firstly. The design variables was set as input and mass, moment of inertia, and unbalance were set as output to complete the simulation. The error evaluation results of the radial basis function response mapping model were obtained as shown in Table 4 by using the interface program to integrate Matlab.

**Table 4:** Error evaluation of the radial basis function response mapping model.

Error evaluation index	Mass /g	Moment of inertia /(g·cm^2^)	Unbalance quantity /(g·cm)
Relative mean error	3.38876e-15	1.7721e-15	2.79205e-15
Relative maximum error	6.0622e-15	4.92746e-15	5.52052e-15
Relative root mean square error	3.60184e-15	2.28878e-15	3.10999e-15
Decisive factor	1	1	1

### Optimization target

Considering the dimensional design range, geometric constraints, and balance performance index range of the key structure parameters of the balanced vibration reduction gear to optimize the variable, a balanced vibration reduction gear constraint model was constructed as follows.


MinF1(X)=m−mwmw-1F2(X)=J−JwJw-1F3(X)=U−UwUw-1s.t.F1(X)≥0;F2(X)≥0;F3(X)≥0;Δmmin≤Δm≤ΔmmaxΔUmin≤ΔU≤ΔUmaxΔJmin≤ΔJ≤ΔJmax;X=x1,x2,⋯x7T;δ≤minixi≤δmaxi (12)

In Formula 12, *F*_*1*_*(X)* is quality objective function, *m* refers to the actual quality, *m*_*w*_ is the target mass. *F*_*2*_*(X)* is objective function of moment of inertia, *J* refers to the actual moment of inertia, *J*_*w*_ is the target moment of inertia. *F*_*3*_*(X)* is unbalance objective function, *J* refers to the actual unbalance, *J*_*w*_ is the target unbalance. X is the key parameter set of structural optimization, *δ*_*i*_ is the i-th optimized variable of the balanced vibration balanced gear, and $\delta_{ij}^{\text{min}}$ and $\delta_{ij}^{\text{max}}$ are the upper and lower bounds of the balanced vibration reduction gear that can be reached by the corresponding processing technology.

## Results and validation

### Optimization results

The nondominated sorting genetic algorithm-II(NSGA-II)was added to the radial basis function mapping model for optimization calculation. In the Isight platform, DOE components, approximation components and numerical optimization components are added from the task components, and the NSGA-II optimization algorithm was selected. After running the NSGA-II multiobjective genetic algorithm program, 525 iterations converge, and the constraints were satisfied. The optimization result is obtained after 240 iterations. The pareto optimal solution obtained is shown in Table 5, which is an NSGA-II multiobjective genetic algorithm optimization scheme.

**Table 5:** NSGA-II multiobjective genetic algorithm optimization results.

Optimization variable	Optimization value	Optimization objective	Optimization value
***x*_1_**	3.000(cm)	*F*_1_(*x*)	0.013
***x*_2_**	2.952(°)	*F*_2_(*x*)	0.172
***x*_3_**	24.185(cm)	*F*_3_(*x*)	0.358
***x*_4_**	0.814(cm)		
***x*_5_**	30.967(°)		
***x*_6_**	0.462(cm)		
***x*_7_**	0.987(cm)		

Considering the impact performance and damping performance of the balanced vibration reduction gear in the actual operation process, this paper used finite element analysis and dynamic analysis software to perform modal analysis and vibration reduction performance on the validation analysis of the optimized gear.

### Validation analysis

The elastic element of the balanced vibration reduction gear designed in this paper adopted rubber material. The rubber material has highly complex nonlinearity, combined with the characteristics of the small deformation of the balanced gear rubber, and the Mooney-Rivlin model was selected as the analysis model of hydrogenated nitrile rubber (HNBR). HNBR is a soft rubber, and the relationship between its hardness HS and material constants C_1_ and C_2_ can be expressed as Formula 13 [30].


$$6C_{1}(1+\frac{C_{2}}{C_{1}})=\frac{15.75+2.15HS}{100-HS}$$ (13)

**Fig 9:**
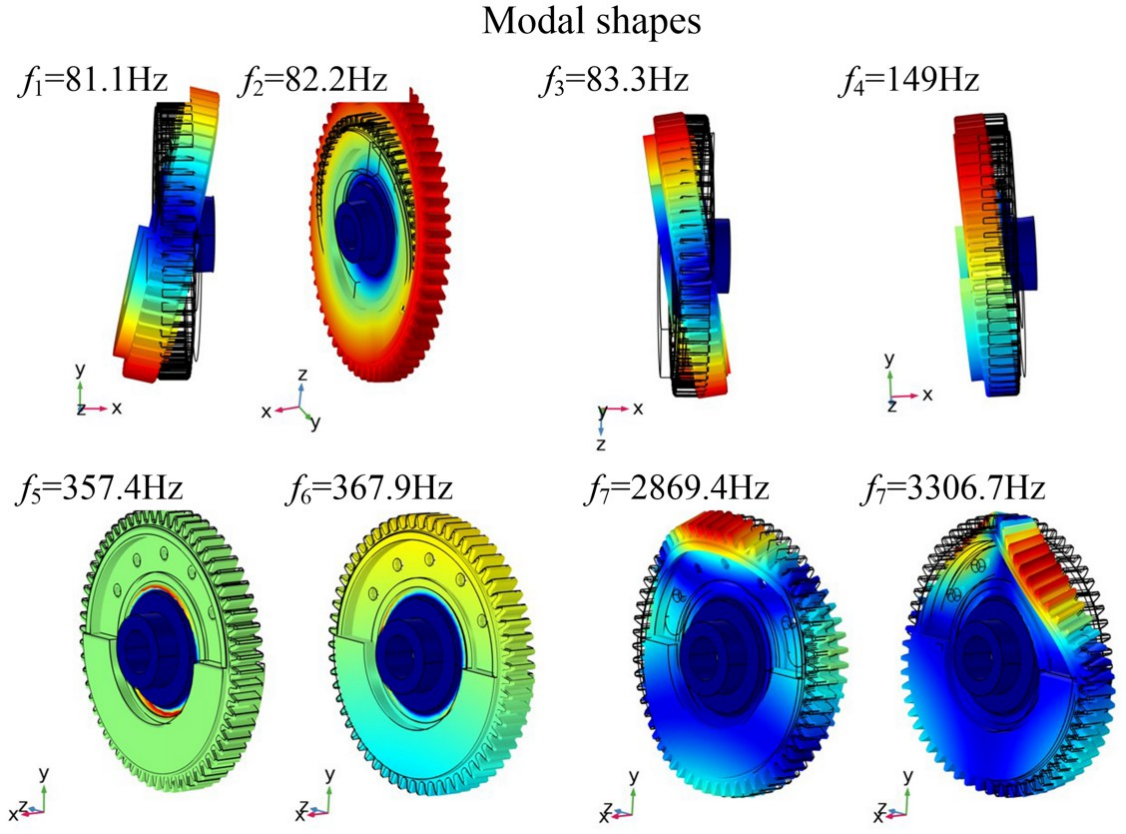
First eight order natural frequency modes.

The modal analysis was carried out based on the commercial multi-physics simulation software COMSOL Mutiphysics. The rubber part of the gear was set as the model parameters as shown in Table 4 to represent the corresponding material properties. For the rest of the gear, the material was set as 50CrMo4 and the density, elastic modulus and Poisson’s ratio are 7.8g/cm^3^, 210 GPa and 0.3 respectively. Modal analysis was carried out at a higher mesh quality. Natural frequencies under different rubber hardness conditions were obtained as shown in Table 6. The modal modes corresponding to different natural frequencies are consistent, and the first eight modes are shown in Fig 9.

**Table 6:** Natural frequency table of balanced gear.

Hardness	Rubber parameters	*f*_1_	*f*_2_	*f*_3_	*f*_4_	*f*_5_	*f*_6_	*f*_7_	*f*_8_
	(MPa)	(Hz)	(Hz)	(Hz)	(Hz)	(Hz)	(Hz)	(Hz)	(Hz)
**HS55**	C10	0.4727	81.1	82.2	83.3	149	357.4	367.9	2869.4
	C01	0.0236							
**HS60**	C10	0.5744	89.4	90.6	91.8	164.2	393.8	405.3	2870
	C01	0.0287							
**HS65**	C10	0.7052	99.0	100.3	101.7	181.8	436.1	448.8	2870.5
	C01	0.0352							
**HS70**	C10	0.8796	110.5	112.0	113.5	203.0	486.6	500.9	2871.2
	C01	0.044							
**HS75**	C10	1.1238	124.9	126.6	128.2	229.2	549.4	565.5	2872.1
	C01	0.0561							
**Rigidity**	/	/	1005.5	1031.2	1639.4	1851.6	1866.5	2434.8	3901.8

Table 6 shows that with increasing modal order, the natural frequencies of the balanced gear and rigid gear gradually increase. Fig 9 shows that as the stiffness of the rubber ring continues to increase, the natural frequencies of the balanced gear continue to increase, but it was far smaller than the rigid gear, so the design of the balanced vibration damping structure is reasonable.

To analyze the influence of the stiffness and damping characteristics of the rubber ring on the gear meshing characteristics and vibration, the assembly model of the optimized balanced gear meshing transmission was established using Solidworks software and imported into Adams to verify its meshing characteristics and virbration reduction characteristics.

In order to ensure the precise transmission of the gear pair, contact constraints were set to simulate the meshing action of the gear pair. Set the damping coefficient as 50, stiffness coefficient as 870000, penetration depth *d* = 0.1mm, nonlinear index as 2.7, static friction coefficient as 0.08, dynamic friction coefficient as 0.05. The difference of dynamic characteristics between reduction gear and rigid gear was studied when the resistance moment is 5N∙m and the driving speed is 1200r/min, 2400r/min and 3600r/min in the paper.

According to the principles of mechanical design, the calculation formulas for the radial stiffness and torsional stiffness of the rubber ring are shown in Formula 14.


$$\left\{\begin{array}{c} K_{l}=\frac{2\pi lG}{\text{ln}\frac{d_{2}}{d_{1}}}\\ K_{T}=\frac{\pi (d_{2}^{4}-d_{1}^{4})G}{32l} \end{array}\right.$$ (14)

**Fig 10:**
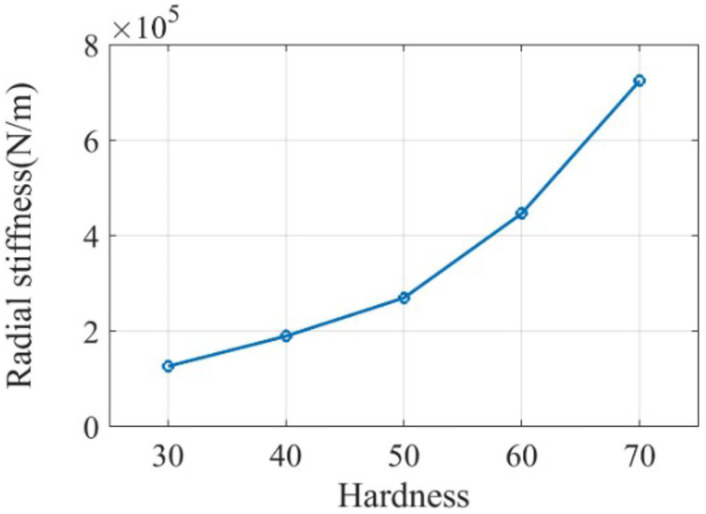
Radial stiffness corresponding to different rubber ring properties.

**Fig 11:**
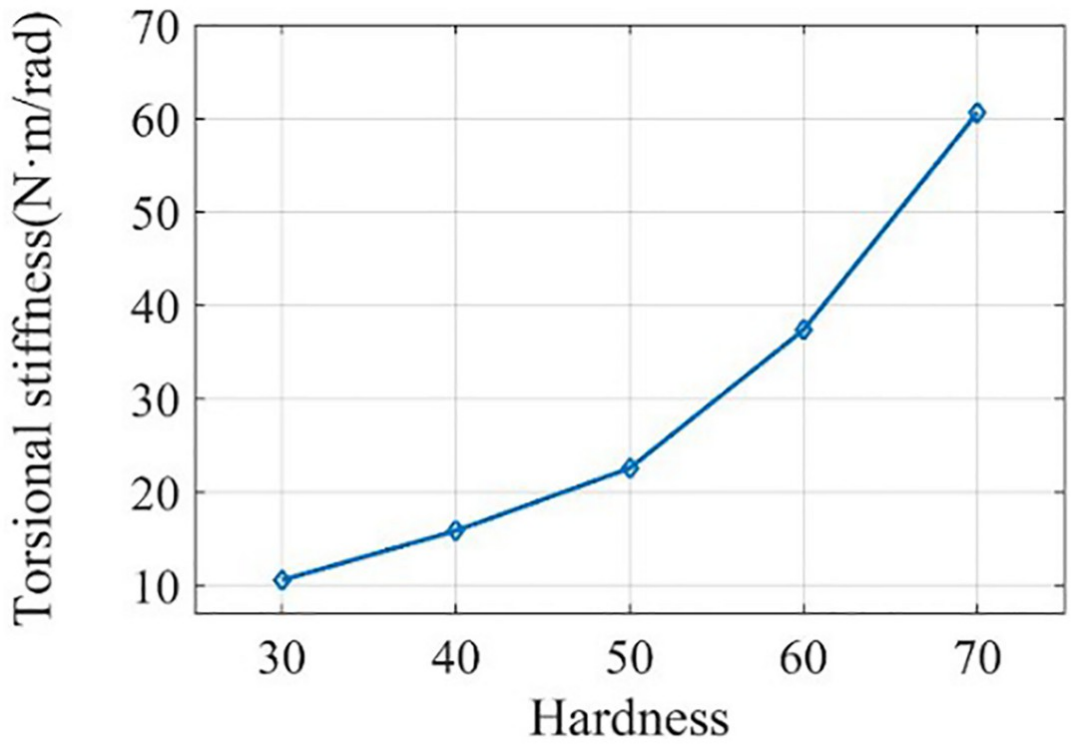
Torsional stiffness corresponding to different rubber ring properties.

Where *K*_l_ is the radial stiffness, *K*_T_ is the torsional stiffness, *d*_2_ is the outer diameter of the rubber ring, *d*_1_ is the inner diameter of the rubber ring, *G* is the shear modulus, and *l* is the thickness of the rubber ring. According to the size of the rubber ring and the value range of the shear modulus, the relationship curve between hardness and stiffness was constructed, as shown in Figs 10 and 11. We mainly focused on the torsional stiffness in the range of 0–100 N·m/rad and the radial stiffness in the range of 10000–1000000 N/m. The parameter setting of the rubber ring stiffness in the simulation parameters was completed in this interval.

**Fig 12:**
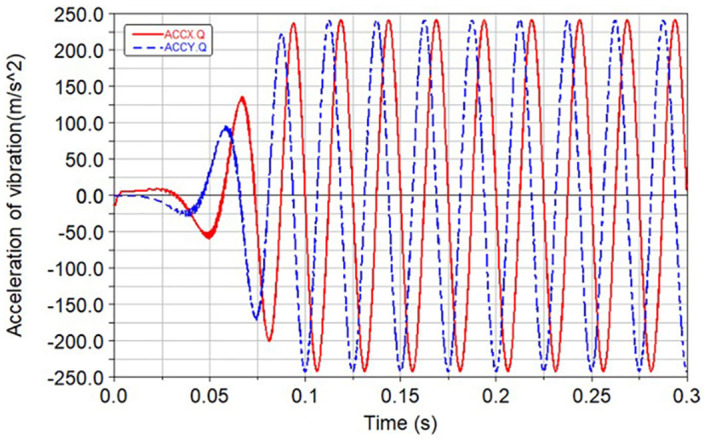
Vibration response during rigid gear transmission.

**Fig 13:**
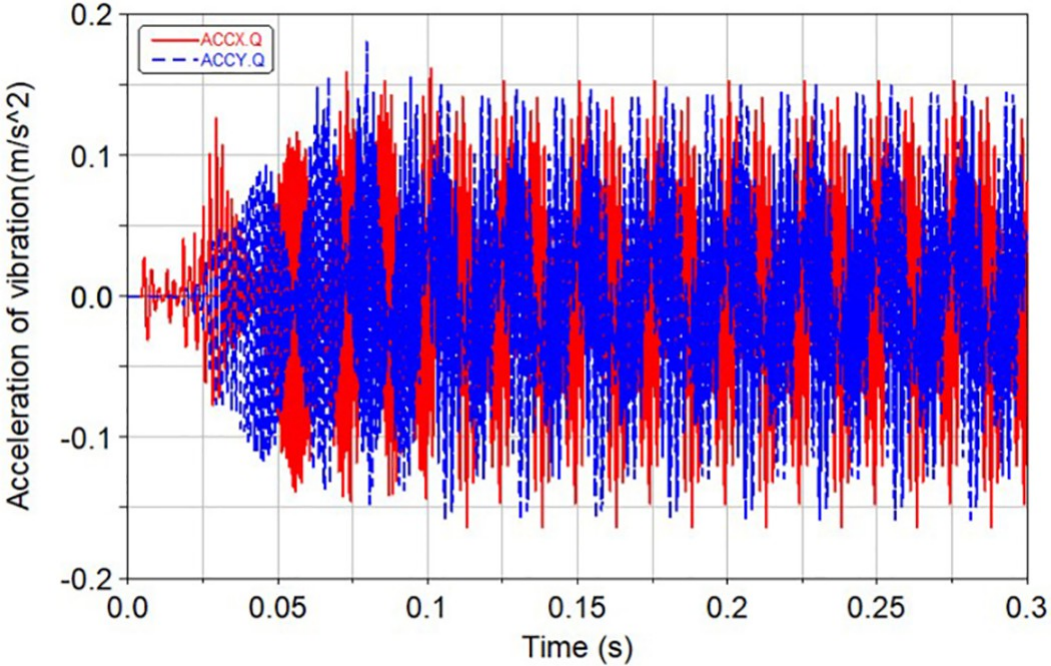
Vibration response during balanced gear transmission.

Based on rigid gears, the influence of different rubber ring stiffness characteristics on the vibration and meshing force is analyzed. The system runs for 0.1 s and enters a stable state. In the case of rigid gear transmission and balanced gear transmission, the vertical and lateral vibration response curves of the balance shaft near the gear are shown in Figs 12 and 13 respectively.

Analyzing the above curve, it could be seen that when the radial stiffness of the rubber ring was selected from 100000 to 200000 N/m and the torsional stiffness is 30 N·m/rad, the vibration damping effect was the best. The comparison results are shown in Table 7. The balanced gear attenuates 99% of the vibration compared to the rigid gear.

**Table 7:** Comparison of vibration response of the old and new system.

Category	Statistical characteristics	X-axis (m/s^2^)	Y-axis (m/s^2^)
Rigid gear	Rms	148.63	148.57
	Max	241.96	241.52
Balanced gear	Rms	0.06	0.06
	Max	0.16	0.18

## Discussion

In summary, based on the DOE and proxy model, the design and optimization method for the gear of three-cylinder engine balanced system proposed in this paper are reasonable and feasible. The flexible mode of the improved balanced vibration reduction gear is not within the range of the gear meshing frequency. The dynamic simulation completed the investigation of the hardness design and damping effect of the rubber ring. Compared with pure rigid gear transmission, the balanced gear designed in this paper can effectively reduce the engine vibration caused by the balanced gear under different speed conditions, and the vibration attenuation reached 99%. However, there are still many areas for improvement in this paper due to the time, equipment and other factors. In the future, we plan to further explore the mechanism of the influence of speed and stiffness on the vibration reduction effect and try to perform physical processing and experimental verification.

## Supporting information

S1 File Calculate coordinate system and optimization result. (PDF)

S2 File Relevant information. (PDF)

## References

[1] Mohammadi S, Ohadi A, and Keshavarz R. Multi-objective optimization of counterweights: a substitute for the balance shaft or mass unbalancing in three-cylinder engines. SAE Int. J. Engines 2018;11(5):557–569. doi: 10.4271/03-11-05-0038

[2] Guan N, Wang A, Gu Y, Xie Z, Zhou M. A novel coaxial balance mechanism for reciprocating piston engines. Appl. Sci. 2021;11: 5647.

[3] Ishikawa M, Nakamura Y, Kodama N, Hosoi H. Development of resin gear balance shaft system for 2AZ-FE engine. Jsae Review. 2002;23(1):27–32. doi: 10.1016/S0389-4304(01)00164-3

[4] G Mathan, J Daniel, P Vishnu, V Satyanarayana. Impact of web and counterweight shape factors on crank shaft design for better strength and optimised geometry. IOP Conference Series: Materials Science and Engineering. 2021;1128(1):012045 1–14.

[5] Trieschmann J, Nagler R, inventors; MITEC Automotive AG, assignee. Gear and balance shaft for a piston engine. United States patent US US20140013888A1. 2014 Jan 16.

[6] Moetakef M A, inventor; Ford Global Technologies LLC, assignee. Counterbalance ounterbalance gear for an engine. United States patent US 20190085763. 2019 Mar 21.

[7] Alessio Courtial, Giuseppe Grioli, Paolo Marocco, Giuliano Sperlinga, inventors; GM Global Technology Operations LLC, assignee. In-line balance shaft system for internal combustion engines. United States patent US 20200063827.2020 Feb 27.

[8] Wen F, Cai GW, Wang HQ, Li YZ. Study on dynamic characteristic of internal parallel moving gears transmission with balance structure. Advanced Materials Research. 2013; 655–657:537–541. doi: 10.4028/www.scientific.net/AMR.655-657.537

[9] Renping S, Jia P, Qi X. 3-D Elastic coupling vibration and acoustical radiation characteristics of cracked gear under elastic support condition. Journal of Vibration and Control. 2017; 23(9): 1548–1568. doi: 10.1177/1077546315596482

[10] Singh OP, Sreenivasulu T, Kannan M. The effect of rubber dampers on engine’s NVH and thermal performance. Applied Acoustics. 2014; 75(1): 17–26. doi: 10.1016/j.apacoust.2013.07.007

[11] Gao F. Complete shaking force and shaking moment balancing of four types of six-bar linkages. Mechanism and Machine Theory. 1989;4(24): 275–287. doi: 10.1016/0094-114X(89)90047-5

[12] Wehrle E, Palomba I, Vidoni R. In-operation structural modification of planetary gear sets using design optimization methods. Mechanisms and Machine Science. 2019; 66:395–405. doi: 10.1007/978-3-030-00365-4_47

[13] Monkova Katarina, Monka Peter, Tkac Jozef, Hricova Romana, Mandulak Dusan. Effect of the weight reduction of a gear wheel on modal characteristics. MATEC Web of Conferences. 2019; 299 (03002):1–6. doi: 10.1051/matecconf/201929903002

[14] Wang S, Wang XL, Wang YR and Ye H. An equivalent damping numerical prediction method for the ring damper used in gears under axial vibration. Symmetry. 2019; 11:1469. doi: 10.3390/sym11121469

[15] Kumar SM, Govindaraj E, Balamurugan D, Daniel F. Design analysis and fabrication of automotive transmission gearbox using hollow gears for weight reduction. Materials Today: Proceedings. 2021; 45: 6822–6832. doi: 10.1016/j.matpr.2020.12.1005

[16] Geng ZB, Li JY, Xiao K, Wang JX. Analysis on the vibration reduction for a new rigid–flexible gear transmission system. Journal of Vibration and Control. 2021; 2: 107754632110132. doi: 10.1177/10775463211013245

[17] Han L, Liu G, Yang XH, Han B. Dimensional and layout optimization design of multistage gear drives using genetic algorithms. Mathematical Problems in Engineering. 2020;1: 3197395. doi: 10.1155/2020/3197395

[18] Kim Jaeseung, Kim Suchul, Sohn Jonghyeon, Moon Sanggon, Lee Geunho. Optimization of gear webs for rotorcraft engine reduction gear train. Journal of the Korean Society for Aeronautical & Space Sciences. 2020; 48(12):953–960. doi: 10.5139/JKSAS.2020.48.12.953

[19] Qi Z, Wang X, Chen W. A new forming method of straight bevel gear using a specific die with a flash. International Journal of Advanced Manufacturing Technology. 2019; 100: 3167–3183. doi: 10.1007/s00170-018-2862-4

[20] Xu L, Bi KM, Gao JF, Xu Y, Zhang C. Analysis on parameter optimization of dampers of long-span double-tower cable-stayed bridges. Structure and Infrastructure Engineering. 2019; 9:1–16. doi: 10.1080/15732479.2019.1703760

[21] Lucay Freddy A., Sales-Cruz Mauricio, Gálvez Edelmira D., Cisternas Luis A. Modeling of the complex behavior through an improved response surface methodology, Mineral Processing and Extractive Metallurgy Review. 2020; 42(5): 285–311. doi: 10.1080/08827508.2020.1728265

[22] Karthik Pandiyan G., Prabaharan T. Optimization of machining parameters on AA6351 alloy steel using response surface methodology (RSM). Materials Today: Proceedings. 2020; 7(33):2686–2689. doi: 10.1016/j.matpr.2020.01.369

[23] Blair R.W., Dunne N.J., Lennon A.B., Menary G.H. Multi-objective optimisation of material properties and strut geometry for poly(L-lactic acid) coronary stents using response surface methodology. PLOS ONE. 2019;14(8):e0218768. doi: 10.1371/journal.pone.0218768 31449528 PMC6709949

[24] Xie ZF, et al. A new closed-form method for inertia force and moment calculation in reciprocating piston engine design. ENCE CHINA Technological Sciences. 2018; 7(9): 1–15. doi: 10.1007/s11431-017-9184-x

[25] Yue C, et al. Unbalance identification of speed-variant rotary machinery without phase angle measurement. Shock and Vibration, 2015; 1–11. doi: 10.1155/2015/934231

[26] Bmha, Amcmc A B. Experimental optimization of the vanes geometry for a variable geometry turbocharger (VGT) using a design of experiment (DOE) approach. Energy Conversion and Management. 2015; 106: 1057–1070. doi: 10.1016/j.enconman.2015.10.040

[27] Namazizadeh Mohammad, Talebian Gevari M, Mojaddam M, Vajdi M. Optimization of the splitter blade configuration and geometry of a centrifugal pump impeller using design of experiment. Journal of Applied Fluid Mechanics. 2020; 13(1): 89–101. doi: 10.29252/jafm.13.01.29856

[28] Morelli M, Bellosta T, Guardone A. Efficient radial basis function mesh deformation methods for aircraft icing. Journal of Computational and Applied Mathematics. 2021; 392(1776): 113492. doi: 10.1016/j.cam.2021.113492

[29] Jin ZY, Li NN, Yan K, Chen JX, Wei DL, Cui ZS, et al. Controlling flow instability in straight spur gear forging using numerical simulation and response surface method. Acta Metallurgica Sinica(English Letters). 2018; 31(1): 82–96. doi: 10.1007/s40195-017-0669-1

[30] Gent A N, Campion R P. Engineering with rubber: how to design rubber components. Hanser Gardner Publications, Carl Hanser Publishers, 2001.

