# Supplementary material for: Structure design method of new balanced vibration reduction gear for the three cylinder engine
Source: PLoS One. 2022 Apr 13;17(4):e0266560. doi: 10.1371/journal.pone.0266560 (PMC9007366; doi:10.1371/journal.pone.0266560)
Supplement: S1 File — (PDF) [file pone.0266560.s001.pdf]

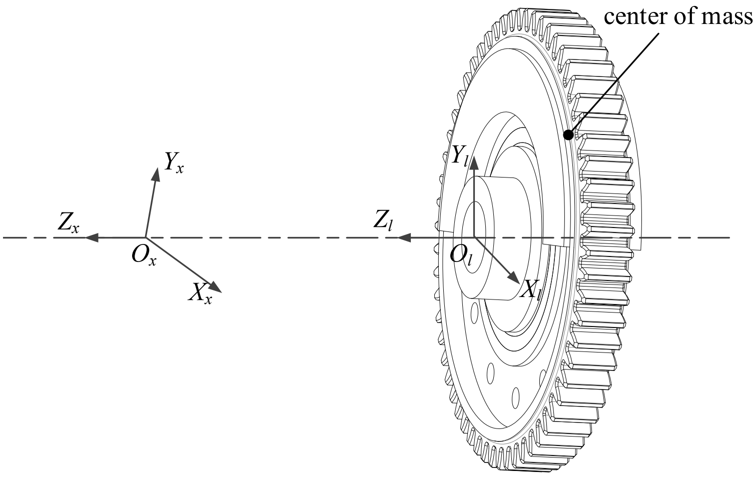

Fig1. Coordinate system for calculating balance characteristic index of balanced damping gear

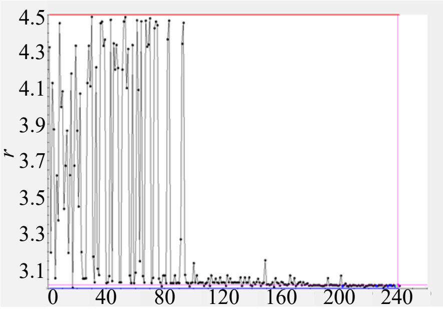

a. Number of iterations of parameter r

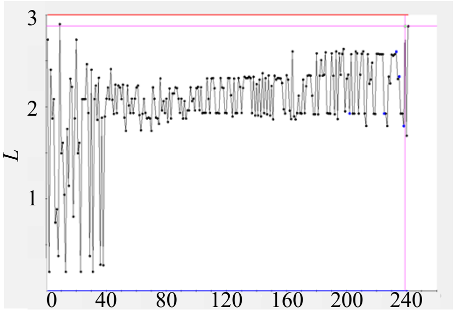

b. Number of iterations of parameter L

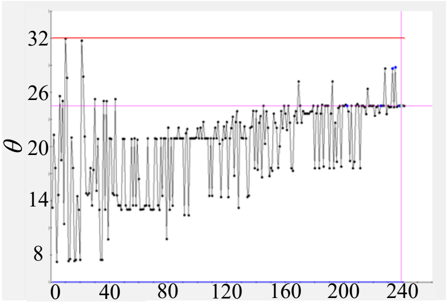

c. Number of iterations of parameter θ

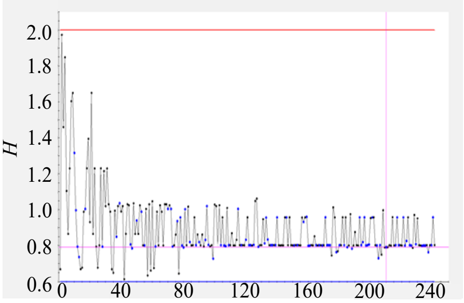

d. Number of iterations of parameter H

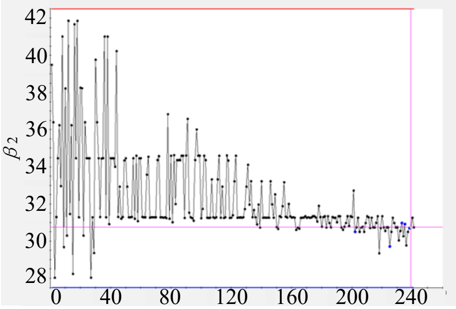

e. Number of iterations of parameter β_2

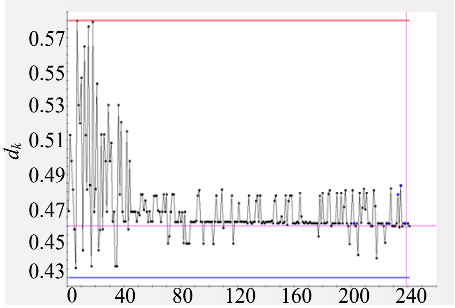

f. Number of iterations of parameter d_k

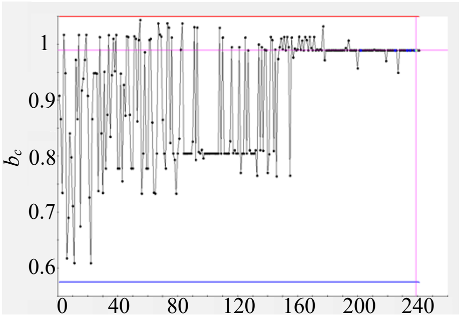

g. Number of iterations of parameter b_c

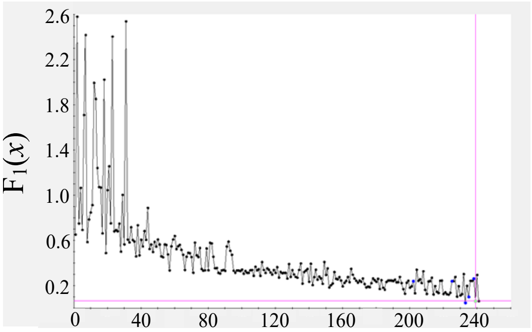

h. Number of iterations of the objective function $F_1(x)$

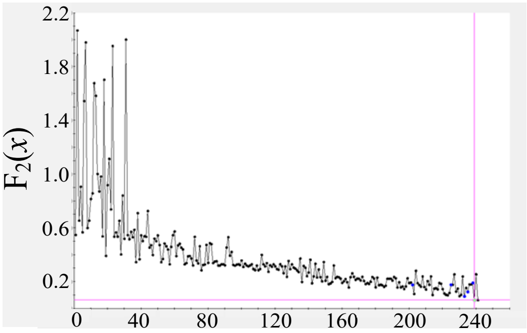

i. Number of iterations of the objective function $F_2(x)$

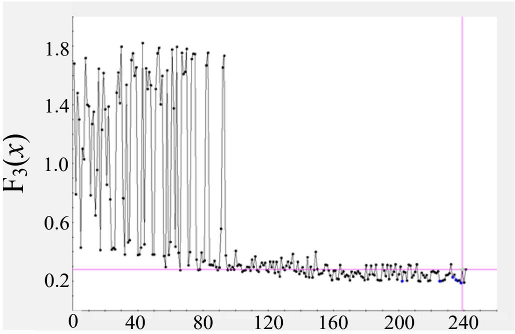

j. Number of iterations of the objective function $F_3(x)$

Fig2. Pareto optimization solution distribution curve based on NSGA-II multi-objective genetic algorithm
