# Supplementary material for: Structure design method of new balanced vibration reduction gear for the three cylinder engine
Source: PLoS One. 2022 Apr 13;17(4):e0266560. doi: 10.1371/journal.pone.0266560 (PMC9007366; doi:10.1371/journal.pone.0266560)
Supplement: S2 File — (PDF) [file pone.0266560.s002.pdf]

Table 1 Orthogonal test results of equilibrium characteristic indexes

test number	Balance performance index			synthesis value
	mass (g)	moment of inertia ($g\cdot cm^2$)	Unbalanced amount ($g\cdot cm$)	
1	483.7943	7338.1371	-20.7806	-0.3928
2	502.3333	4855.2327	81.8509	4.9977
3	466.1427	4490.1181	-52.8604	-2.2802
4	483.3386	7448.4526	-0.8305	0.6841
5	489.3599	7119.6449	-31.1922	-0.9598
6	558.0551	6948.5152	175.0388	10.1519
7	483.9673	7452.0409	-0.8305	0.6847
8	461.7349	4130.4704	-71.3991	-3.2980
9	520.7116	7869.3800	101.2718	6.2148
10	476.5490	6942.0132	-70.9702	-3.1140
11	539.7137	8422.8342	149.2768	8.8352
12	474.9017	5705.3329	-39.8279	-1.5092
13	477.2771	5799.6381	-37.5095	-1.3781
14	461.5266	2924.8981	-76.3798	-3.6303
15	461.5266	2924.8981	-76.3798	-3.6303
16	512.2667	5171.1104	98.6964	5.9260
17	527.9902	8096.4464	113.7472	6.9018
18	462.4747	4137.3242	-69.6740	-3.2045
19	515.7060	7836.5670	107.0628	6.5206
20	513.3481	3682.7883	48.9846	3.1774
21	479.4023	7256.0574	-23.8512	-0.5650
22	480.0233	7376.0362	-14.4555	-0.0537
23	480.0233	7308.4323	-17.1729	-0.2032
24	481.8220	7404.2297	-10.6639	0.1527
25	479.7564	7314.1939	-16.5931	-0.1719
26	463.5359	4474.0697	-52.8604	-2.2829
27	484.2846	7288.6384	-24.0925	-0.5729

Table 2 Results of balance characteristic index of 36 groups of response design of balanced vibration reduction gear

**scheme**	***r* (cm)**	**θ (°)**	***L* (cm)**	***H* (cm)**	**d_k (cm)**	**β_2 (°)**	**b_c (cm)**	**mass (g)**	**moment of inertia ($g\cdot cm^2$)**	**unbalanced amount ($g\cdot cm$)**
**1**	4.29	68.86	0.76	0.78	0.48	60.38	0.55	589.72	8999.55	437.04
**2**	3.38	50.63	3.00	0.59	0.38	105.95	0.72	591.84	8996.25	449.81
**3**	4.06	31.39	1.90	0.87	0.49	143.92	0.99	598.12	9080.83	460.18
**4**	3.51	61.77	2.39	0.05	0.45	145.44	0.82	580.15	8785.94	403.44
**5**	4.16	14.18	1.29	0.09	0.42	142.41	0.76	580.99	8817.17	407.76

**6**	4.35	9.11	1.82	1.38	0.45	95.32	0.80	593.84	9042.69	452.32
**7**	3.13	56.71	2.47	0.85	0.51	116.58	0.86	600.32	9074.36	473.00
**8**	3.36	46.58	0.08	0.71	0.44	37.59	0.64	633.65	9569.52	541.60
**9**	4.31	35.44	0.15	0.39	0.43	99.87	0.97	584.05	8817.96	408.18
**10**	4.23	36.46	1.03	1.05	0.39	146.96	0.86	597.98	9123.81	458.80
**11**	3.82	10.13	2.24	1.06	0.40	131.77	0.62	625.04	9610.99	567.80
**12**	4.50	43.54	1.86	0.96	0.50	36.08	0.81	577.64	8636.98	375.66
**13**	4.41	44.56	2.92	0.62	0.44	137.85	0.77	581.69	8829.82	410.92
**14**	3.19	49.62	1.22	1.31	0.47	42.15	0.87	647.38	9687.82	599.63
**15**	3.59	8.10	0.61	1.24	0.41	83.16	0.94	665.54	10159.7	639.51
**16**	4.03	67.85	2.43	1.40	0.49	92.28	0.77	593.89	9023.80	456.16
**17**	3.11	28.35	2.81	1.21	0.45	78.61	0.65	630.64	9570.05	591.46
**18**	3.06	70.89	2.13	0.50	0.46	108.99	0.56	590.61	8991.89	446.72
**19**	3.84	30.38	0.91	1.29	0.53	75.57	0.63	637.81	9801.81	583.50
**20**	4.44	58.73	2.05	1.22	0.39	92.28	0.67	583.11	8863.79	418.66
**21**	3.85	16.20	2.89	0.90	0.52	107.47	0.71	608.47	9304.44	512.38
**22**	3.27	75.95	0.49	0.98	0.50	96.84	0.69	609.81	9222.72	497.26
**23**	3.32	25.32	2.96	0.12	0.47	64.94	0.79	583.69	8825.75	412.91
**24**	3.61	39.49	2.62	1.35	0.41	110.51	0.93	618.43	9399.49	539.05
**25**	4.01	52.66	2.32	0.07	0.39	80.13	0.93	579.20	8735.38	394.36
**26**	3.76	40.51	1.48	0.69	0.45	90.76	0.77	605.40	9212.63	487.74
**27**	3.55	71.90	1.06	0.00	0.48	63.42	0.65	580.06	8797.08	403.47
**28**	3.48	73.92	1.56	1.26	0.42	60.38	0.58	606.20	9232.66	494.36
**29**	3.67	27.34	2.73	0.94	0.49	55.82	1.00	617.08	9479.43	495.45
**30**	3.70	77.97	0.11	0.64	0.41	118.10	0.80	597.16	9069.94	452.60
**31**	3.02	46.58	0.95	1.12	0.38	102.91	0.75	653.24	9862.23	623.83
**32**	3.17	80.00	2.09	0.53	0.43	72.53	0.90	583.53	8796.57	408.88
**33**	4.04	29.37	0.23	0.23	0.52	89.24	0.74	587.83	8916.21	426.46
**34**	3.78	11.14	0.76	0.16	0.44	71.01	0.53	590.09	8995.41	439.26
**35**	3.97	78.99	0.57	0.67	0.49	46.71	0.89	588.87	8869.18	416.28
**36**	4.10	74.94	2.77	0.35	0.44	74.05	0.62	582.53	8852.09	415.85
